# Reduced Use of Emergency Care and Hospitalization in Patients with Traumatic Brain Injury Receiving Acupuncture Treatment

**DOI:** 10.1155/2013/262039

**Published:** 2013-07-18

**Authors:** Chun-Chuan Shih, Hsun-Hua Lee, Ta-Liang Chen, Chin-Chuan Tsai, Hsin-Long Lane, Wen-Ta Chiu, Chien-Chang Liao

**Affiliations:** ^1^School of Chinese Medicine for Post-Baccalaureate, I-Shou University, Kaohsiung 84001, Taiwan; ^2^Department of Neurology, Shuang-Ho Hospital, Taipei Medical University, New Taipei 235, Taiwan; ^3^Department of Anesthesiology, Taipei Medical University Hospital, 252 Wuxing Street, Taipei 110, Taiwan; ^4^Health Policy Research Centre, Taipei Medical University Hospital, Taipei 110, Taiwan; ^5^School of Medicine, College of Medicine, Taipei Medical University, Taipei 110, Taiwan; ^6^Graduate Institute of Injury Prevention and Control, Taipei Medical University, Taipei 110, Taiwan

## Abstract

*Background*. Little research exists on acupuncture treatment's effect on patients with traumatic brain injury (TBI). *Methods*. Using Taiwan's National Health Insurance Research Database, we conducted a cohort study to compare the use of emergency care and hospitalization in TBI patients with and without acupuncture treatment in the first year after TBI. The adjusted relative risks (RRs) and 95% confidence intervals (CIs) of high use of emergency care and hospitalization associated with acupuncture treatment were calculated in multivariate Poisson regression models with generalized estimating equation. *Results*. The means of medical visits of emergency care and hospitalization were lower in TBI patients with acupuncture treatment than in those without acupuncture treatment. After adjustment, acupuncture treatment was associated with decreased risk of high emergency care visits (beta = −0.0611, *P* = 0.0452) and hospitalization (beta = −0.0989, *P* < 0.0001). The RRs of high medical visits and expenditure for hospitalization associated with acupuncture treatment were 0.62 (95% CI = 0.50–0.76) and 0.66 (95% CI = 0.53–0.83), respectively. *Conclusion*. Patients with TBI who receive acupuncture treatment have reduced the use of emergency care and hospitalization in the first year after injury. The mechanisms of effects of acupuncture on TBI warrant further investigations.

## 1. Introduction

Traumatic brain injury (TBI), a common injury across every age group and both sexes [1–4], causes disability and death worldwide. It causes 1.1 million emergency visits, 235,000 hospitalisations, and 50,000 deaths in the United States every year [5, 6]. The socioeconomic impacts and burden of diseases for disability following TBI are potentially long term or lifelong [2–5, 7–14]. While the epidemiology, natural history, risk factors, and outcomes of TBI have been established [1–17], TBI treatment and rehabilitation are still crucial problems of global concern.

Despite the remarkable developments in modern Western medicine (a medical system included physicians, surgeons, and other healthcare professionals (such as nurses, pharmacists, and therapists) to treat symptoms, illness, and diseases using biochemical drugs, radiation, or surgery in the clinics or hospitals) in modern times [18], there is great public interest in complementary and alternative medicine, such as acupuncture [19]. Acupuncture is an important part of traditional Chinese medicine (TCM) widely used in Taiwan [20–25] and other Asian and Western countries [26–29]. Acupuncture's effectiveness has been widely confirmed [30–32], and almost a quarter of Taiwan's people (23%) used acupuncture between 1996 and 2002 [25].

There has been no population-based epidemiological study with comprehensive study design documenting the effectiveness of acupuncture treatment on patients with TBI. A recent investigation reported that acupuncture had a beneficial effect on cognition and on perception of sleep or sleep quality for patients with TBI [33]. However, that study was limited by small sample size, poor study design, and inadequate adjustment. It was also reported that acupuncture may reduce medical expenditure for patients with chronic neck pain or angina [34, 35]. Based on the findings from previous reports, our study sought to investigate the effectiveness of acupuncture treatment on patients with TBI in a nationwide population-based cohort study with matching procedure by propensity score and multivariate adjustment.

## 2. Methods

### 2.1. Study Design and Population

**Figure 1:**
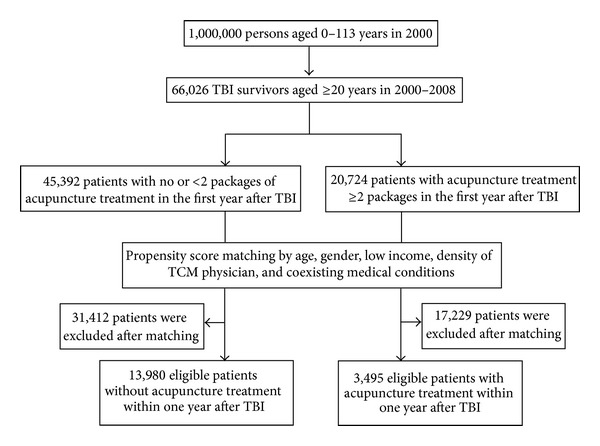
Selecting eligible TBI patients with and without acupuncture treatment (TBI: traumatic brain injury; TCM: traditional Chinese medicine).

Taiwan's National Health Research Institutes has documented all medical claims for insured beneficiaries since 1996 and provides these as the National Health Insurance Research Database for public access. With patient identification numbers scrambled, data files can be secured to protect patient privacy. Information available for this study included gender, birth date, disease codes, health care rendered, medicines prescribed, diagnoses for admissions and discharges, and medical institutions and physicians providing services. Details were described in our previous studies [1–3, 20]. From a longitudinal cohort population-based database of a randomly selected one million insured subjects, we identified persons aged ≥20 years with newly diagnosed TBI who made visits for medical care in 2000–2008 as our eligible study patients (Figure 1). In order to confirm that all patients with TBI in our study were incident cases, only new-onset TBI cases were included in this study; people with previous medical records of TBI within five years before the index date were excluded. The diagnosis of TBI was validated in previous studies [1–3]. Overall, we identified 66,026 new-onset TBI patients aged ≥20 years; we excluded mortality cases after TBI within one year. Among new-onset TBI patients, 3495 had used at least two packages (one package included 6 treatments) of acupuncture treatment after injury within the first year. For each patient with TBI who had acupuncture treatment, we randomly selected 4 subjects without acupuncture treatment from patients with TBI as controls matched by age, sex, low income, density of TCM physicians, and coexisting medical conditions (such as hypertension, mental disorder, diabetes mellitus, stroke, ischemic heart disease, hyperlipidemia, migraine, and epilepsy). We conducted our analysis using propensity score-matched pair procedure. Nearest-neighbor algorithm was applied to construct matched pairs, assuming that the proportion of 0.95 to 1.0 is perfect. We followed TBI patients with and without acupuncture treatment for one year and collected their corresponding medical records.

The outcome of this study is the high use (including frequency and medical expenditure) of emergency care and hospitalization for people with a diagnosis of TBI in the first year after this injury. This study used matching procedure with propensity score to minimize the difference of sociodemographic factors and coexisting medical conditions between TBI patients with and without acupuncture treatment. This study's objective is to investigate whether acupuncture treatment reduces the use frequency and medical expenditure for emergency care and hospitalization for patients with TBI.

### 2.2. Criteria and Definition

We defined TBI according to the International Code of Diseases, Ninth Edition, Clinical Modification (ICD-9-CM 800–805, 850–854). Coexisting medical conditions included hypertension (ICD-9-CM 401–405), mental disorders (ICD-9-CM 290–319), diabetes (ICD-9-CM 250), stroke (ICD-9-CM 430–438), ischemic heart disease (ICD-9-CM 410–414), hyperlipidemia (ICD-9-CM 272.0–272.4), migraine (ICD-9-CM 346), and epilepsy (ICD-9-CM 345). From individuals' health reimbursement claims, regular renal dialysis (including hemodialysis and/or peritoneal dialysis) was also considered a coexisting medical condition for TBI patients in this study. As Taiwan has 359 townships and city districts, we calculated the population density (persons/km^2^) for each of these administrative units. Based on population density, these areas were stratified into tertiles of low, moderate, and high urbanization [20–22]. In this study, TCM physicians were defined as physicians licensed by Taiwan's Department of Health who practiced TCM in the legal clinics or hospitals. We calculated the density of TCM physicians (TCM physicians/10,000 persons) using the number of TCM physicians per 10,000 residents for each administrative unit. The first, second, and third tertiles were considered as areas with low, moderate, and high physician density.

We classified the frequency and medical expenditure for emergency care and hospitalization into quintiles [36]. Patients in the highest quintile of medical visits were defined as those having high visits for emergency care or hospitalization. Patients in the highest quintile of medical expenditure (calculated in US dollars) were defined as having high medical expenditure for emergency care or hospitalization.

### 2.3. Statistical Analysis

**Figure 2:**
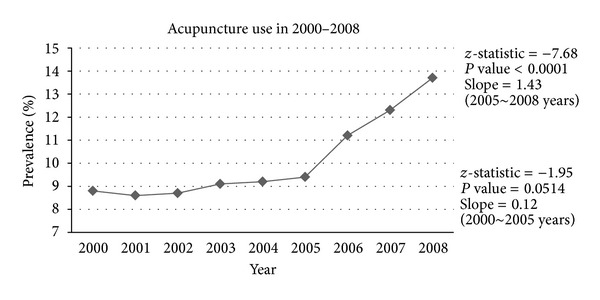
Annual prevalence of use of acupuncture treatment by patients with traumatic brain injury (by Cochran-Armitage trend test).

The annual prevalence of the utilization of acupuncture treatment by patients with TBI was calculated between 2000 and 2008. The Cochran-Armitage trend tests were analyzed for the prevalence of acupuncture use in 2000–2005 and 2005–2008 (Figure 2). With matching procedure of propensity score, we matched factors including age, sex, low income, density of TCM physicians, diabetes, hypertension, hyperlipidemia, mental disorders, ischemic heart disease, stroke, migraine, and epilepsy between TBI patients with and without acupuncture treatment. The mean and standard deviation of visits and medical expenditure of emergency care and hospitalization were compared with unpaired *t*-tests between patients who had TBI with and without acupuncture treatment. We performed multiple linear regressions to analyze the use of emergency care and hospitalization associated with acupuncture treatment. The adjusted relative risks (RRs) and 95% confidence intervals (CIs) of high medical expenditure of emergency care and hospitalization associated with acupuncture treatment were calculated in the multivariate Poisson regression analyses with generalized estimating equation. All analyses were performed using Statistical Analysis Software version 9.1 (SAS Institute Inc., Cary, NC, USA). A two-sided probability value of <0.05 was considered significant.

## 3. Results

The increased trend of utilization of acupuncture treatment for patients with TBI is shown in Figure 1. The slopes of increased use of acupuncture treatment in 2000–2005 and 2005–2008 were 0.12 (*P* = 0.0514) and 1.43 (*P* < 0.0001), respectively. After matching procedure with propensity score, there was no significant difference in age, sex, low income, density of TCM physician, diabetes, hypertension, hyperlipidemia, mental disorders, ischemic heart disease, stroke, migraine, and epilepsy between TBI patients with and without acupuncture treatment (Table 1).

Compared with TBI patients without acupuncture treatment, patients with TBI who underwent acupuncture treatment had lower medical expenditure for emergency care (44.0 ± 154.2 versus 54.6 ± 520.0 US dollars, *P* = 0.04) and hospitalization (517.2 ± 2588.3 versus 941.0 ± 5099.7 US dollars, *P* < 0.0001) within the first year after TBI (Table 2). The means of medical visits for emergency care (0.48 ± 1.21 versus 0.56 ± 2.02 visits, *P* = 0.006) and hospitalization (0.29 ± 0.85 versus 0.39 ± 0.98 visits, *P* < 0.0001) were also lower in TBI patients with acupuncture treatment than in TBI patients without acupuncture treatment.

In patients with TBI, acupuncture treatment was associated with reduced visits for emergency care (beta = −0.0611, *P* = 0.0452) and hospitalization (beta = −0.0989, *P* < 0.0001) during the postinjury year (Table 3). The decreased medical expenditure for hospitalization (beta = −11772.47, *P* < 0.0001) for patients with TBI was also associated with acupuncture treatment.

After adjustment for age, sex, low income, TCM physician density, diabetes mellitus, hypertension, hyperlipidemia, mental disorder, migraine, epilepsy, ischemic heart disease, and stroke (Table 4), TBI patients with acupuncture treatment had decreased risk of high medical visits for hospitalization (RR = 0.62, 95% CI = 0.50–0.76) in the first year compared with TBI patients without acupuncture treatment. The relative risks of high medical expenditure for hospitalization associated with acupuncture treatment in the first year were 0.66 (95% CI = 0.53–0.83).

## 4. Discussion

Using Taiwan's nationwide insurance database, we reported an increasing trend of acupuncture use for patients with TBI between 2000 and 2008. Using comprehensive study design with the matching procedure of propensity score, our study discovered decreased use of medical resources for emergency and hospitalization for patients in their first post-TBI years after receiving acupuncture treatment. The present study is the first to report the effects of acupuncture treatment for patient with TBI using population-based investigation from Taiwan.

### 4.1. Potential Confounding Factors

Age and sex determined the medical conditions and complications for people after their traumatic brain injuries. The utilization of TCM or acupuncture was also associated with age and sex [20–24]. In general, males and older people had worse outcomes after TBI. In contrast, females and young people were more likely to use acupuncture treatment. In addition, low income is a factor associated with TBI risk [1–3]. Density of TCM physicians and low income were related factors for utilization of acupuncture [21]. To investigate the effects of acupuncture treatment on TBI in the current study, we used propensity score to match the effects of age, sex, low income, and density of TCM physicians between TBI patients with and without acupuncture treatment. To accurately estimate post-TBI high medical visits and expenditure for emergency care and hospitalization, residual confounding effects were also controlled in the multivariate linear regressions and Poisson regression models with generalized estimating equation.

Hypertension, mental disorders, diabetes, ischemic heart disease, stroke, and epilepsy were risk factors for TBI [1]. Epilepsy and migraine were identified as common complications after TBI [2]. Compared with non-TBI patients, patients with TBI had increased risk of stroke [4]. To eliminate confounding effects from these TBI-related coexisting medical conditions, we used matching procedure with propensity score and multivariate adjustment to control hypertension, mental disorders, diabetes, stroke, ischemic heart disease, hyperlipidemia, migraine, and epilepsy [1–3].

### 4.2. Possible Explanations

Insomnia is a frequent problem in patients with TBI. A pilot intervention study found that acupuncture has a beneficial effect on perception of sleep or sleep quality and on cognition in a small sample of TBI patients [33]. Acupuncture treatment was thus suggested as an economical substitute for some medical services and pharmaceuticals; this was a finding of some importance to insurers, healthcare practitioners, and policy makers [27].

In our study, patients with TBI who received acupuncture treatment had reduced use of emergency care and hospitalization in the first year after injury compared with those without acupuncture treatment. We propose two possible explanations. First, the effect of acupuncture treatment on TBI patients was due to biological effects from acupuncture. The stimulation of acupuncture may lead to activation of frontal-limbic-striatal brain regions, with the pattern of neural activity somewhat different for each acupuncture point [19]. A study from Taiwan also showed the cerebral hemodynamic responses of acupuncture stimulation modes and implied that its mechanism was not only based upon afferent sensory information processing but also had the hemodynamic property altered during external stimulation [37]. Acupuncture intervention involves complex modulations of temporal neural response and involves multiple levels of differential activities of a wide range of brain networks [38]. It can enhance the poststimulation spatial extent of resting brain networks to include antinociceptive memory and affective brain regions. This modulation and sympathovagal response may relate to acupuncture analgesia and other potential therapeutic effects [39]. Second, patients with TBI who choose acupuncture treatment may have better knowledge, attitudes, and practices regarding physical rehabilitation, which we believe could reduce adverse events after TBI.

### 4.3. Study Limitation

This study has some limitations. First, as we used retrospective medical claims data from health insurance that lacked detailed patient information on lifestyle as well as physical, psychiatric, and laboratory examinations, we were unable to differentiate whether these factors influenced the effects of acupuncture treatment on TBI patients. 

Second, as our study used ICD-9-CM codes claimed by physicians for the TBI without clarifying the severity of disease, some of those higher utilization and expenditure might be associated with more severe conditions. Our study would have been improved if we had Glasgow coma score information [7]. Third, this study was limited by the data provided by insurance claims which might be underestimated because very minor TBI might not seek medical treatment. In addition, our study could not show the acupuncture points used in patient care due to the limited information from National Health Insurance Research Database.

## 5. Conclusion

Using Taiwan's National Health Insurance Research Database, our comprehensive designed retrospective cohort study suggested that patients with TBI who receive acupuncture treatment had reduced use of emergency care and hospitalization in the first year of injury. Our findings implicate that acupuncture treatment may reduce symptoms and illness from major complications for patients with TBI. However, these assumptions need future prospective studies to provide information on the effects of acupuncture treatment on post-TBI mortality and complications, such as stroke, mental disorders, epilepsy, migraine, and fracture. Clinical trials are also needed to clarify the acupuncture points that best address TBI patient needs. The mechanisms of activation and deactivation and their effects on TBI warrant further investigations as well.

## Figures and Tables

**Table 1:** Matched characteristics and coexisting medical conditions for patients who had traumatic brain injury with and without acupuncture treatment.

	Acupuncture treatment				
	No (*N* = 13980)		Yes (*N* = 3495)		*P* value
	*n*	(%)	*n*	(%)	
Age, years					1.00
20–29	3024	(21.6)	756	(21.6)	
30–39	2464	(17.6)	616	(17.6)	
40–49	2596	(18.6)	649	(18.6)	
50–59	2356	(16.9)	589	(16.9)	
60–69	1632	(11.7)	408	(11.7)	
70–79	1448	(10.4)	362	(10.4)	
≥80	460	(3.3)	115	(3.3)	
Mean ± SD	47.34 ± 18.03		47.39 ± 17.80		0.96
Gender					1.00
Female	7980	(57.1)	1995	(57.1)	
Male	6000	(42.9)	1500	(42.9)	
Low income					1.00
No	13944	(99.7)	3486	(99.7)	
Yes	36	(0.3)	9	(0.3)	
Density of TCM physician					1.00
Low	1600	(11.4)	400	(11.4)	
Moderate	5304	(37.9)	1326	(37.9)	
High	7076	(50.6)	1769	(50.6)	
Coexisting medical conditions					
Hypertension	2364	(16.9)	591	(16.9)	1.00
Mental disorder	2128	(15.2)	532	(15.2)	1.00
Diabetes mellitus	1064	(7.6)	266	(7.6)	1.00
Stroke	784	(5.6)	196	(5.6)	1.00
Ischemic heart disease	664	(4.8)	166	(4.8)	1.00
Hyperlipidemia	556	(4.0)	139	(4.0)	1.00
Migraine	200	(1.4)	50	(1.4)	1.00
Epilepsy	88	(0.6)	22	(0.6)	1.00

SD: standard deviation; TCM: traditional Chinese medicine.

**Table 2:** Visits and medical expenditure of emergency care and hospitalization between patients with and without acupuncture treatment in the first year after traumatic brain injury.

	Acupuncture treatment		*P* value
	No (*N* = 13980)	Yes (*N* = 3495)	
Frequency of visits, mean ± SD			
Emergency care	0.54 ± 1.73	0.47 ± 1.16	0.01
Hospitalization	0.38 ± 0.99	0.29 ± 0.89	<0.0001
Medical expenditure, mean ± SD			
Emergency care	46.6 ± 154.9	42.3 ± 145.8	0.12
Hospitalization	872.6 ± 5717.6	480.2 ± 2426.6	<0.0001

SD: standard deviation.

**Table 3:** Multiple linear regression analysis of medical visits and expenditure for emergency care and hospitalization for patients having traumatic brain injury with and without acupuncture treatment in the first year after injury.

	Acupuncture treatment*	
	Beta	*P* value
Medical visits		
Emergency care	−0.06	0.0452
Hospitalization	−0.10	<0.0001
Medical expenditure		
Emergency care	−130.44	0.1260
Hospitalization	−11772.47	<0.0001

*Adjusted for age, gender, low income, TCM physician density, diabetes mellitus, hypertension, hyperlipidemia, mental disorder, migraine, epilepsy, ischemia heart disease, and stroke.

TCM: traditional Chinese medicine.

**Table 4:** Risks of high medical visits and of high emergency care and hospitalization for patients with and without acupuncture treatment in the first year after traumatic brain injury.

	No acupuncture			Acupuncture treatment*		
	Cases	RR	(95% CI)	Cases	RR	(95% CI)
High medical visits						
Emergency care	800	1.00	(Reference)	17	0.88	(0.75–1.02)
Hospitalization	640	1.00	(Reference)	99	0.62	(0.50–0.76)
High medical expenditure						
Emergency care	707	1.00	(Reference)	172	0.97	(0.83–1.14)
Hospitalization	506	1.00	(Reference)	84	0.66	(0.53–0.83)

*Adjusted for age, gender, low income, TCM physician density, diabetes mellitus, hypertension, hyperlipidemia, mental disorder, migraine, epilepsy, ischemia heart disease, and stroke.

CI: confidence interval; RR: relative risk; TCM: traditional Chinese medicine.

## References

[1] Liao CC, Chang HC, Yeh CC, Chiu WT, Chen TL (2012). Risk and outcomes for traumatic brain injury in patients with mental disorders. *Journal of Neurology, Neurosurgery, and Psychiatry*.

[2] Yeh CC, Chen TL, Hu CJ, Chiu WT, Liao CC (2013). Risk of epilepsy after traumatic brain injury: a retrospective population-based cohort study. *Journal of Neurology, Neurosurgery, and Psychiatry*.

[3] Liao CC, Chang HC, Yeh CC, Chou YC, Chiu WT, Chen TL (2012). Socioeconomic deprivation and associated risk factors of traumatic brain injury in children. *The Journal of Trauma and Acute Care Surgery*.

[4] Chen Y-H, Kang J-H, Lin H-C (2011). Patients with traumatic brain injury: population-based study suggests increased risk of stroke. *Stroke*.

[5] Yates PJ, Williams WH, Harris A, Round A, Jenkins R (2006). An epidemiological study of head injuries in a UK population attending an emergency department. *Journal of Neurology, Neurosurgery and Psychiatry*.

[6] Centers for Disease Control and Prevention (2007). Rates of hospitalization related to traumatic brain injury—nine states, 2003. *Morbidity and Mortality Weekly Report*.

[7] Kung W-M, Tsai S-H, Chiu W-T (2011). Correlation between Glasgow coma score components and survival in patients with traumatic brain injury. *Injury*.

[8] Chen Y-H, Chiu W-T, Chu S-F, Lin H-C (2011). Increased risk of schizophrenia following traumatic brain injury: a 5-year follow-up study in Taiwan. *Psychological Medicine*.

[9] Chiu W-T, Huang S-J, Tsai S-H (2007). The impact of time, legislation, and geography on the epidemiology of traumatic brain injury. *Journal of Clinical Neuroscience*.

[10] Chiu W-T, Huang S-J, Hwang H-F (2006). Use of the WHOQOL-BREF for evaluating persons with traumatic brain injury. *Journal of Neurotrauma*.

[11] Chen Y-H, Keller JJ, Kang J-H, Lin H-C (2012). Association between traumatic brain injury and the subsequent risk of brain cancer. *Journal of Neurotrauma*.

[12] Kang J-H, Lin H-C (2012). Increased risk of multiple sclerosis after traumatic brain injury: a nationwide population-based study. *Journal of Neurotrauma*.

[13] Chen Y-H, Chiu W-T, Chu S-F, Lin H-C (2010). Increased risk of schizophrenia following traumatic brain injury: a 5-year follow-up study in Taiwan. *Psychological Medicine*.

[14] Chen CJ, Wu CH, Liao YP (2012). Working memory in patients with mild traumatic brain injury: functional MR imaging analysis. *Radiology*.

[15] Patel HC, Bouamra O, Woodford M, King AT, Yates DW, Lecky FE (2005). Trends in head injury outcome from 1989 to 2003 and the effect of neurosurgical care: an observational study. *The Lancet*.

[16] Fleminger S, Ponsford J (2005). Long term outcome after traumatic brain injury. *British Medical Journal*.

[17] Yu W-Y, Chen C-Y, Chiu W-T, Lin M-R (2011). Effectiveness of different types of motorcycle helmets and effects of their improper use on head injuries. *International Journal of Epidemiology*.

[18] Chi C, Lee J-L, Lai J-S, Chen C-Y, Chang S-K, Chen S-C (1996). The practice of Chinese medicine in Taiwan. *Social Science and Medicine*.

[19] Quah-Smith I, Sachdev PS, Wen W, Chen X, Williams MA (2010). The brain effects of laser acupuncture in healthy individuals: an FMRI investigation. *PloS oNE*.

[20] Liao CC, Lin JG, Tsai CC (2012). An investigation of the use of traditional Chinese medicine in stroke patients in Taiwan. *Evidence-Based Complementary and Alternative Medicine*.

[21] Shih C-C, Liao C-C, Su Y-C, Tsai C-C, Lin J-G (2012). Gender differences in traditional chinese medicine use among adults in Taiwan. *PLoS ONE*.

[22] Shih C-C, Liao C-C, Su Y-C, Yeh TF, Lin J-G (2012). The association between socioeconomic status and traditional Chinese medicine use among children in Taiwan. *BMC Health Services Research*.

[23] Shih C-C, Su Y-C, Liao C-C, Lin J-G (2010). Patterns of medical pluralism among adults: results from the 2001 National Health Interview Survey in Taiwan. *BMC Health Services Research*.

[24] Shih C-C, Lin J-G, Liao C-C, Su Y-C (2009). The utilization of traditional Chinese medicine and associated factors in Taiwan in 2002. *Chinese Medical Journal*.

[25] Chen F-P, Kung Y-Y, Chen T-J, Hwang S-J (2006). Demographics and patterns of acupuncture use in the Chinese population: the Taiwan experience. *Journal of Alternative and Complementary Medicine*.

[26] Chao MT, Tippens KM, Connelly E (2012). Utilization of group-based, community acupuncture clinics: a comparative study with a nationally representative sample of acupuncture users. *Journal of Alternative and Complementary Medicine*.

[27] Bonafede M, Dick A, Noyes K, Klein JD, Brown T (2008). The effect of acupuncture utilization on healthcare utilization. *Medical Care*.

[28] Robinson TW (2012). Western acupuncture in a NHS general practice: anonymized 3-year patient feedback survey. *Journal of Alternative and Complementary Medicine*.

[29] Guimarães SB, Da Silva AH, Braga JM (2008). Patterns of acupuncture practice and acupoint usage in brazil: the Fortaleza experience. *JAMS Journal of Acupuncture and Meridian Studies*.

[30] Witt C, Brinkhaus B, Jena S (2005). Acupuncture in patients with osteoarthritis of the knee: a randomised trial. *The Lancet*.

[31] Manheimer E, Zhang G, Udoff L (2008). Effects of acupuncture on rates of pregnancy and live birth among women undergoing in vitro fertilisation: systematic review and meta-analysis. *British Medical Journal*.

[32] Melchart D, Streng A, Hoppe A (2005). Acupuncture in patients with tension-type headache: randomised controlled trial. *British Medical Journal*.

[33] Zollman FS, Larson EB, Wasek-Throm LK, Cyborski CM, Bode RK (2012). Acupuncture for treatment of insomnia in patients with traumatic brain injury: a pilot intervention study. *Journal of Head Trauma Rehabilitation*.

[34] Willich SN, Reinhold T, Selim D, Jena S, Brinkhaus B, Witt CM (2006). Cost-effectiveness of acupuncture treatment in patients with chronic neck pain. *Pain*.

[35] Ballegaard S, Johannessen A, Karpatschof B, Nyboe J (1999). Addition of acupuncture and self-care education in the treatment of patients with severe angina pectoris may be cost beneficial: an open, prospective study. *Journal of Alternative and Complementary Medicine*.

[36] Liao CC, Shen WW, Chang CC, Chang H, Chen TL (2013). Surgical adverse outcomes in patients with schizophrenia: a population-based study. *Annals of Surgery*.

[37] Hsieh CW, Wu JH, Hsieh CH, Wang QF, Chen JH (2011). Different brain network activations induced by modulation and nonmodulation laser acupuncture. *Evidence-Based Complementary and Alternative Medicine*.

[38] Bai L, Tian J, Zhong C (2010). Acupuncture modulates temporal neural responses in wide brain networks: evidence from fMRI study. *Molecular Pain*.

[39] Dhond RP, Yeh C, Park K, Kettner N, Napadow V (2008). Acupuncture modulates resting state connectivity in default and sensorimotor brain networks. *Pain*.

